# Expression of Cyr61 in ApoE^−/−^ mice with chronic unilateral renal artery ligation

**DOI:** 10.1038/s41598-021-81646-1

**Published:** 2021-02-11

**Authors:** Alokkumar S. Pathak, Mauricio Rojas, George A. Stouffer

**Affiliations:** 1 McAllister Heart Institute, University of North Carolina, Chapel Hill, NC USA; 2 Division of Cardiology, University of North Carolina, Chapel Hill, NC 27599-7075 USA

**Keywords:** Cardiovascular biology, Circulation

## Abstract

Cyr61 is a member of the CCN family of proteins that is expressed in atherosclerotic lesions and regulated by angiotensin II. It is unknown whether renal artery stenosis (RAS) increases Cyr61 expression. Male ApoE^−/−^ mice were randomized to surgically induced RAS, RAS + treatment with either irbesartan, aliskiren or amlodipine or sham-surgery. RAS resulted in increased plasma angiotensin II levels, a mild, sustained increase in systolic blood pressure and increased aortic lipid deposition compared to sham-surgery. Surgically induced RAS led to the formation of atheroma in the infrarenal aorta and there was consistent and intense staining for Cyr61 within the atheroma. Treatment with irbesartan, aliskiren and amlodipine were associated with decreased aortic lipid deposition and decreased staining for Cyr61 in aortic atheroma. Serum levels of Cyr61 were not increased in mice or humans with RAS. In summary, Cyr61 expression in aortic atheroma but not serum is increased by RAS in ApoE^−/−^ mice and is reduced by agents that lower blood pressure.

## Introduction

Patients with hemodynamically significant renal artery stenosis (RAS) are at an increased risk of cardiac death, myocardial infarction and stroke^1–4^. Clinical outcomes in this condition are dismal with survival in published studies being approximately 60% at 4 years^1–4^. Therapeutic options are limited as there are no specific pharmaceutical interventions for RAS and three large, randomized trials did not show any improvement in survival with percutaneous revascularization^1–3^.

Cyr61 (also known as CCN1) is a cysteine-rich, heparin-binding, secreted protein that is a member of the CCN family of “matricellular” signaling molecules which regulate cellular responses via interactions with integrin receptors in a cell-type and context-specific manner^5^. There are several lines of evidence suggesting that Cyr61 might play a role in progression of atherosclerosis in patients with RAS. First, Cyr61 is expressed at high levels in arteriosclerotic arteries of humans and ApoE^−/−^ mice whereas expression is minimal in normal arteries^6–9^. Second, vascular expression of Cyr61 is increased after mechanical or drug-induced arterial injury and inhibition of Cyr61in a rat carotid artery balloon injury model significantly suppresses smooth muscle cell (SMC) proliferation and neointimal hyperplasia after injury^8,10–12^. Third, Cyr61 stimulates proliferation and migration of cultured SMC in a dose-dependent manner^10,13^. Lastly, Cyr61 expression is regulated by angiotensin II (Ang-II)^6,14^. Since the pioneering studies of Goldblatt and colleagues, it has been recognized that unilateral RAS is associated with increased serum levels of Ang-II^15^. Specifically, intraperitoneal injection of Ang II increased expression of Cyr61 transcripts in the aorta of mice and treatment of rat aortic SMC with Ang II directly stimulated Cyr61 mRNA expression via an angiotensin type 1 receptor mechanism^6^.

Since mortality rates in obstructive RAS remain persistently high and there is a compelling need for novel therapeutics based on a better understanding of atherogenic mechanisms, the purpose of the present studies was to determine the effects of unilateral renal artery ligation (RAL) with or without pharmacological inhibition of the renin-angiotensin system on vascular and serum expression of Cyr61 in ApoE^−/−^ mice.

## Methods

### Renal artery ligation, blood pressure measurements, tissue harvesting and immunohistochemistry

Surgery and Laser-Doppler imaging were performed as previously described on 18-week-old male ApoE^−/−^ mice (C57BL/6 background) purchased from the Jackson Laboratory (Bar Harbor, ME) who were fed a standard chow diet (Purina, Certified Rodent Chow 5001) and had free access to water^16^. Briefly, the mice were anesthetized and a vertical incision made in the midline of the back. The right renal artery was exposed and a tapered 32 or 33 gauge needle positioned on top of the artery. The vessel was ligated by tying the sutures down firmly around the needle and the vessel and then removing the needle. Blood flow through the renal artery was then assessed by using a 10 MHz pulsed Doppler probe. Systolic blood pressure was measured prior to surgery and then at 15-day intervals using a computerized, noninvasive, tail-cuff system (Hatteras Instruments, Cary NC) which has been validated for reproducibility in acclimatized mice^17^. Ten measurements were made at the same time each day for five consecutive days for each animal. These blood pressures were averaged and reported as occurring on the last day of measurement. Animals were habituated to the device for a week before blood pressures were obtained. Mice that didn’t survive for at least 7 days after surgery were excluded from the study. Blood collection was obtained immediately after concluding blood pressure measurements at 15 day intervals; in brief a similar pattern was followed throughout the study allowing mice to recover for 10 days post blood collection, commencing blood pressure readings 11 days after each blood draw and then measuring blood pressure for five consecutive days followed by another blood collection. Total cholesterol levels were measured using reagents in kit form purchased from Sigma (St. Louis, MO).

The mice were euthanized 90 days after surgery and 4% paraformadehyde in phosphate buffered saline infused for 10 min at a pressure of 100 mm Hg. After tissue fixation, aortic tissue was resected from the aortic valve to the iliac bifurcation and adventitial tissue was carefully removed. The distal one-third of the abdominal aorta was embedded in paraffin blocks, sectioned and stained with Masson's trichrome stain or rabbit anti-Cyr61 polyclonal antibodies raised against a synthetic peptide conjugated to KLH derived from within residues 350–450 of Human Cyr61 (Abcam, Cambridge MA). Lipid deposition in the aorta was assayed using 0.3% Oil-red-O solution as described by Weiss et al.^18^ All mice studies were approved by the UNC Institutional Animal Care and Use Committee.

The studies on mice were performed in accordance with the Animal Research: Reporting of In Vivo Experiments (ARRIVE) guidelines.

### Measurement of plasma levels of angiotensin II

Plasma angiotensin II concentrations were determined using a commercially available Radioimmunoassay kit (Phoenix Pharmaceuticals Inc., Burlingame, California, USA) according to the manufacturer’s instructions. Plasma samples prepared from blood collected in EDTA tubes at various time points via tail-bleed were assayed in duplicates. Gamma-Counter was utilized to measure counts per minute of the pellets. Results were interpreted by natural log transformation of the data and then compared to a standard curve^16^.

### Measurement of serum levels of Cyr61 from mice and patient samples

Mouse serum Cyr61 levels were measured in serum pre-surgery and then at 15, 30 and 90 days after surgery using a commercially available ELISA kit (DRG International Inc., Springfield NJ) according to the manufacturer's instructions.

Patients enrolled in two clinical studies approved by the UNC Institutional Review Board evaluating biomarkers and who had a determination of renal anatomy were identified and stored samples were analyzed for Cyr61 using a commercially available ELISA kit (R&D Systems, Inc., Minneapolis, MN) according to the manufacturer's instructions. One of the studies was designed to analyze biomarkers of contrast-induced nephropathy in high-risk patients undergoing coronary angiography and the other study examined biomarkers associated with the level of coronary artery disease in patients undergoing coronary angiography for chest pain^19^. Renal anatomy assessment was within 6 months of blood sampling and was by direct angiography (n = 22), CT angiography (n = 23) or Doppler ultrasound evaluation (n = 10).

### Data analysis and statistics

Normally distributed data are presented as mean ± SD. Comparison between groups was done via Student’s T-test, Analysis of Variance, One Way Repeated Measures ANOVA or Kruskal–Wallis One Way Analysis of Variance (for non-Normally distributed continuous variables) followed by Holm-Sidak method or Dunn’s Multiple Range test (if groups were unequal in size). Mantel–Haenszel Chi-Square was used to analyze differences between categorical variables. Differences were considered significant at a p-value of ≤ 0.05.

## Results

### Renal artery ligation resulted in decreased renal blood flow and increased plasma Ang II levels

Eighty-nine ApoE^−/−^ mice were randomly assigned to partial unilateral renal artery ligation (RAL; n = 24), RAL + treatment with irbesartan (n = 17), RAL + treatment with aliskiren (n = 15), RAL + treatment with amlodipine (n = 17) or sham surgery (n = 16). Treatment with irbesartan (10 mg/kg/day), aliskiren (10 mg/kg/day) or amlodipine (6 mg/kg/day) was via drinking water and begun two days prior to surgery. Irbesartan and aliskiren inhibit the renin-angiotensin system via different mechanisms—irbesartan is an inhibitor of the Ang II type 1 (AT1) receptor whereas aliskiren is an inhibitor of renin. Both drugs have been shown to reduce atherosclerosis progression in the 2 kidney, 1 clip mouse model of RAL^20^. Amlodipine is a member of the dihydropyridine class of calcium channel inhibitors. Prior studies have shown that aliskiren decreases plasma renin activity (PRA) whereas irbesartan and amlodipine are associated with increases in PRA^20^.

**Figure 1:**
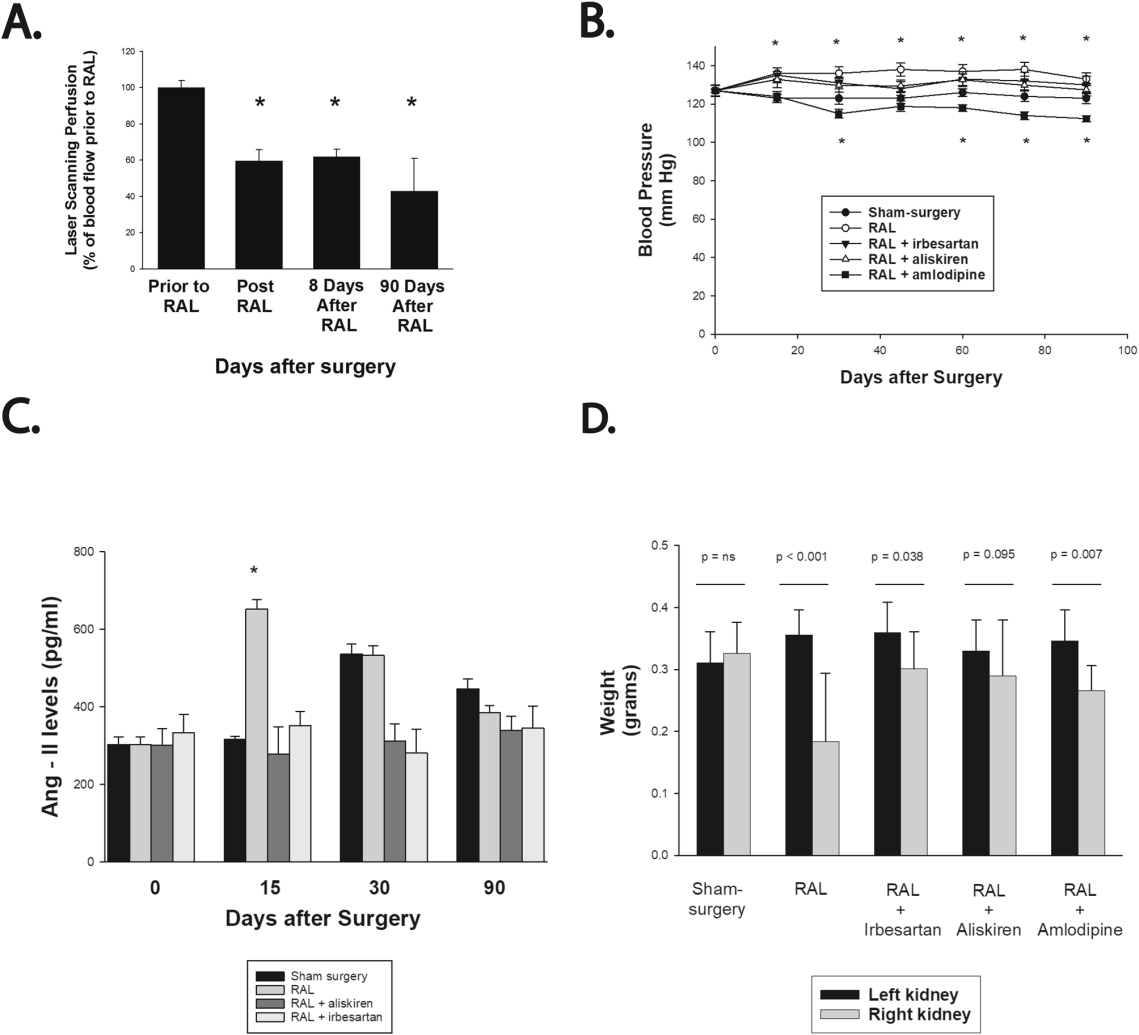
Renal perfusion and physiologic effects following unilateral renal artery ligation. (**A**) Laser-Doppler imaging results at different time points after RAL. (**B**) Systolic blood pressures measured on five successive days both prior to surgery and then 15, 30, 45, 60, 75 and 90 days after surgery (**C**) Plasma Ang II levels measured at various time points. (**D**) Weight (mean ± SEM) of right and left kidneys at the time of sacrifice. [*p < 0.05 compared to sham-surgery].

Partial ligation of the right renal artery resulted in a reduction of blood flow to the right kidney to an average of 60 ± 6% of baseline flow with the reduction in blood flow being maintained when measured at 8 and 90 days after RAL (Fig. 1A). RAL elicited a mild, sustained increase in systolic blood pressure with statistically significant blood pressure elevation apparent throughout the course of the experiment (*p* < 0.05 compared to blood pressure prior to surgery; Fig. 1B). In contrast, sham-surgery had no effect on blood pressure during the duration of the experiment (*p* = 0.87). Treatment with irbesartan or aliskiren at the doses used in this study prevented the hypertensive response to RAL whereas amlodipine had a hypotensive effect. Plasma angiotensin II levels were increased at day 15 in the RAL group and this effect was completely inhibited by treatment with aliskiren or irbesartan (Fig. 1C). Angiotensin II levels were numerically higher at 30 and 90 days in the sham-surgery group compared to baseline but these differences were not statistically significant. Angiotensin II levels were not measured in the RAL + amlodipine group as previous studies have shown that angiotensin II levels were similar in ApoE^−/−^ mice with surgically induced RAL compared to ApoE^−/−^ mice with surgically induced RAL who were treated with amlodipine when measured 4 weeks after surgery^20^.

At the time of sacrifice 90 days after surgery, the right kidney was significantly smaller than the left kidney in the RAL, RAL + irbesartan and the RAL + amlodipine groups but there was no difference in weight between the left and right kidneys in the sham-operated or RAL + aliskiren groups (Fig. 1D). Body weight of the mice was similar in all groups at the time of sacrifice (data not shown). RAL had no effect on plasma cholesterol levels when measured 7 or 21 days after surgery (5.4 ± 0.6 mmol/l at baseline; 5.5 ± 0.4 mmol/l in sham-surgery group and 5.9 ± 0.8 mmol/l in chronic RAL group 7 days after surgery, *p* = 0.42 and 5.7 ± 0.1 mmol/l in sham-surgery group and 6.1 ± 0.6 mmol/l in chronic RAL group 21 days after surgery, *p* = 0.26).

### Cyr61 is expressed in atheroma in mice with RAL and expression is reduced by treatment with irbesartan, aliskiren or amlodipine

Chronic RAL led to the formation of atheroma in the infrarenal aorta as seen in prior studies^16^. Levels of Cyr61 in atheroma were detected using a rabbit polyclonal antibody against a synthetic peptide derived from a proprietary sequence within residues 350–450 of human Cyr61 conjugated to KLH. This antibody has been shown to bind mouse Cyr61 with high specificity in immunohistochemical studies.

**Figure 2:**
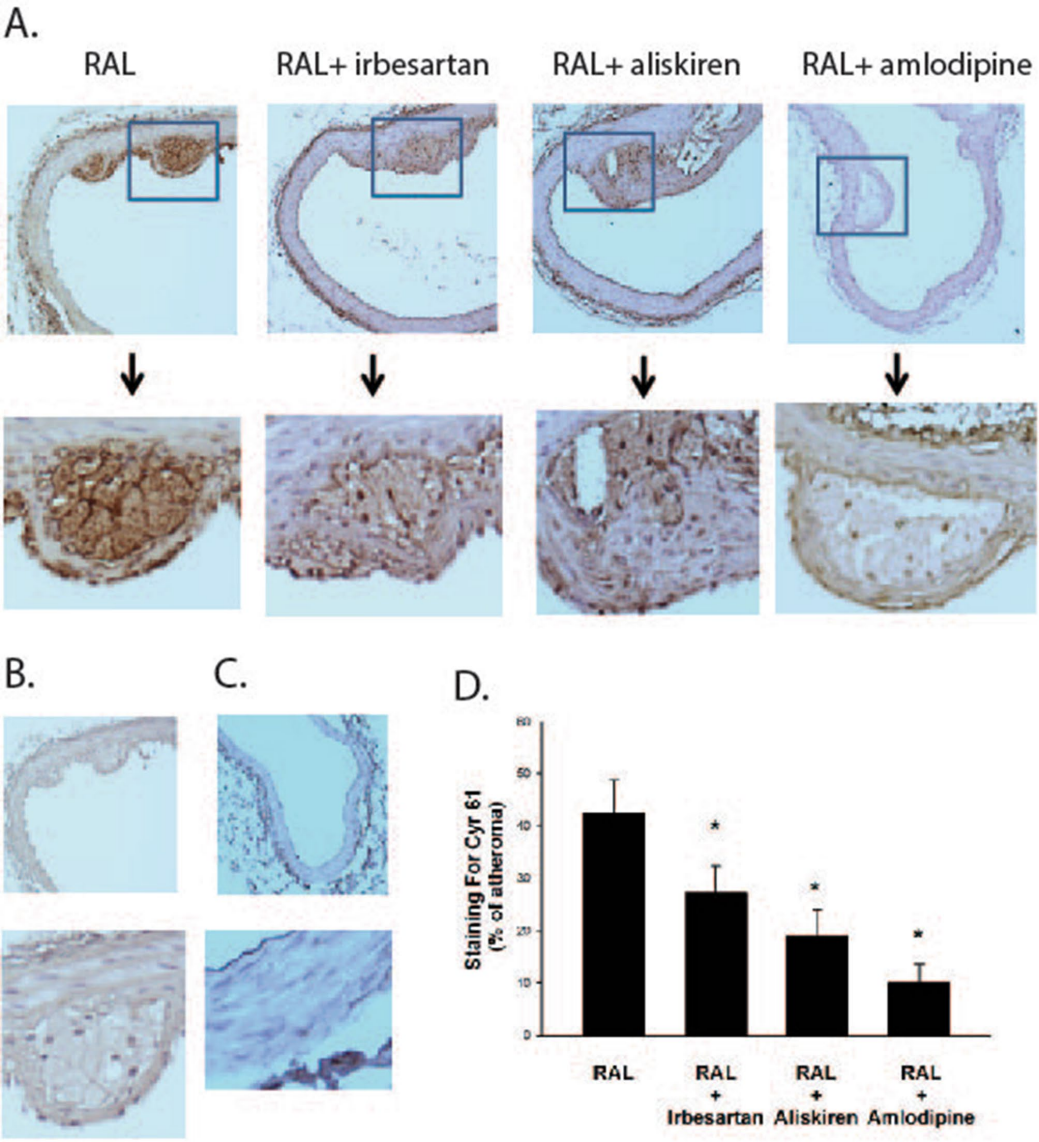
Cyr61 expression in aortic atheromas from ApoE^−/−^ mice with unilateral renal artery ligation. Representative sections from the distal one-third of the descending aorta of ApoE^−/−^ mice from various groups stained with anti-Cyr61 antibody (**A**) or no primary antibody (as a control, **B**). A representative aortic section from a mouse that underwent sham-surgery stained with anti-Cyr61 antibody in shown in panel (**C**). There were no atheroma in mice that underwent sham-surgery. The area of atheroma that stained for Cyr61 is quantified in panel D [*p < 0.05 vs RAL group].

In the RAL group, there was consistent and intense staining for Cyr61 within the atheroma (Fig. 2A) as compared to no staining of the atheroma with a control antibody (Fig. 2B). There were no atheroma in mice in the sham-surgery group and minimal staining for Cyr61 in the artery wall (Fig. 2C). In the RAL + irbesartan, RAL + aliskiren and RAL + amlodipine groups the staining within atheroma was more patchy and diffuse than in the RAL group. Overall there was 35% less staining for Cyr61 in atheromas in the RAL + irbesartan group, 57% less staining for Cyr61 in RAL + aliskiren group and 76% less staining for Cyr61 in RAL + amlodipine group compared to the RAL group (all comparisons to RAL group were *p* < 0.05; Fig. 2D).

### Treatment with irbesartan, aliskiren or amlodipine reduced lipid deposition in the descending aorta and aortic arch of mice with RAL

**Figure 3:**
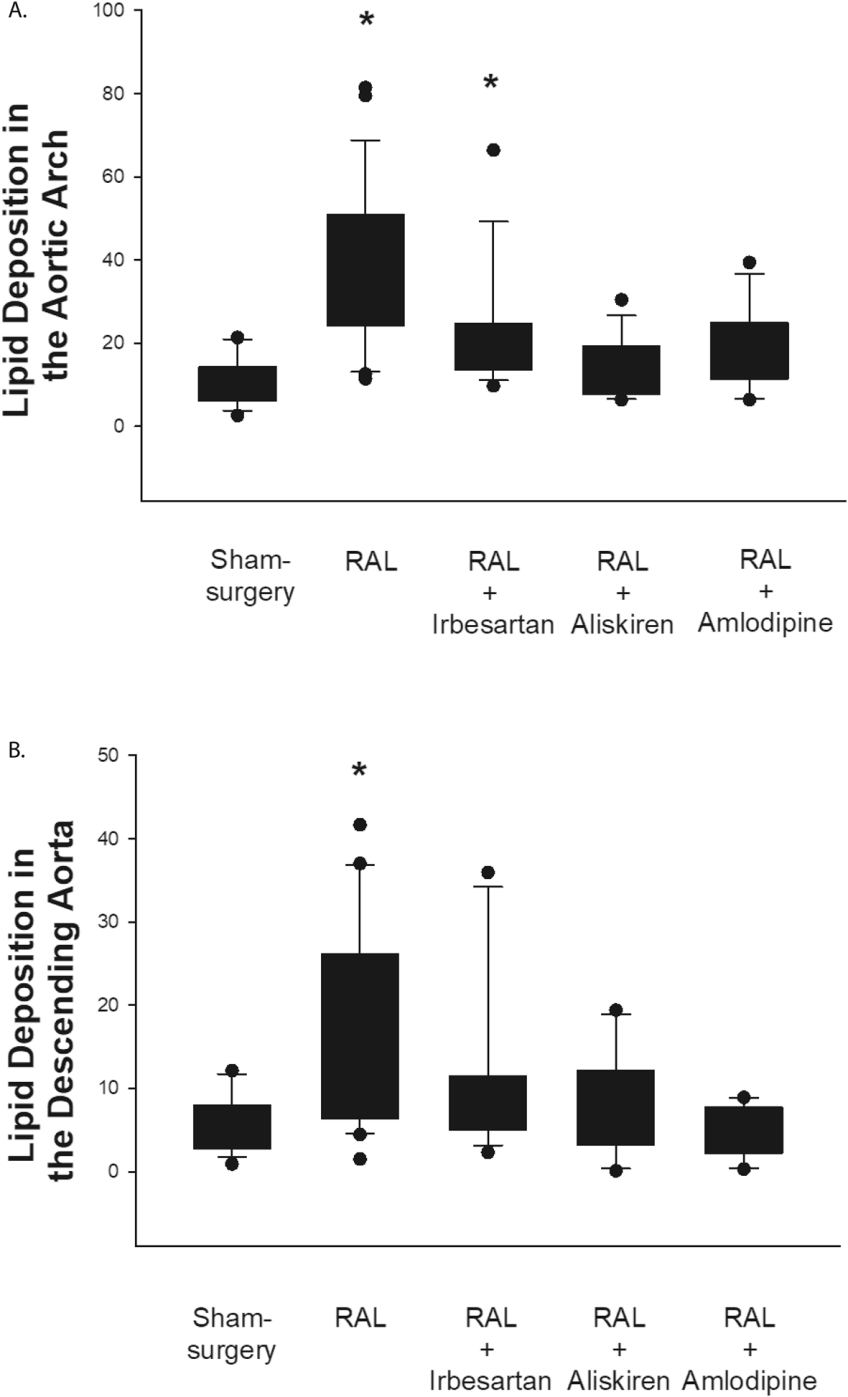
Lipid deposition in the descending aorta and aortic arch following renal artery ligation. The area of the aortic arch (**A**) or descending aorta (**B**) that stained with Oil-red-O as a percentage of total area for all mice in each of the groups. [*p < 0.05 vs sham-surgery].

To determine whether treatment with irbesartan, aliskiren or amlodipine influenced the development of atherosclerotic lesions at doses that decreased Cyr61 expression in atheroma, lipid deposition in the aortic arch and suprarenal descending aorta were measured 90 days after surgery using Oil-red-O staining. Chronic RAL resulted in an approximate threefold increase in lipid deposition in the aortic arch compared to sham-surgery (33.2% [24.3, 50.8] versus 11.6% [6.1, 14.3]; *p* = 0.01) (Fig. 3A). Treatment with aliskiren (16.0% [7.7, 19.3]) or amlodipine (19.1% [11.4, 24.9]) reduced the increase in aortic arch lipid deposition seen with chronic RAL whereas treatment with irbesartan (22.0% [13.5, 24.6]) reduced the amount of lipid deposition in the aortic arch in mice with chronic RAL but the difference did not reach statistical significance.

Chronic RAL resulted in an approximate twofold increase in lipid deposition in the suprarenal descending aorta compared to the sham-surgery group (10.2% [6.4, 26.0] versus 5.0% [2.8, 7.8]; *p* = 0.01) (Fig. 3B)^21,22^. Treatment with irbesartan, aliskiren or amlodipine prevented the increase in descending aorta lipid deposition seen with chronic RAL (Fig. 3B).

### Serum levels of CYR61 were not increased in mice with unilateral RAL or in humans with obstructive RAS

**Figure 4:**
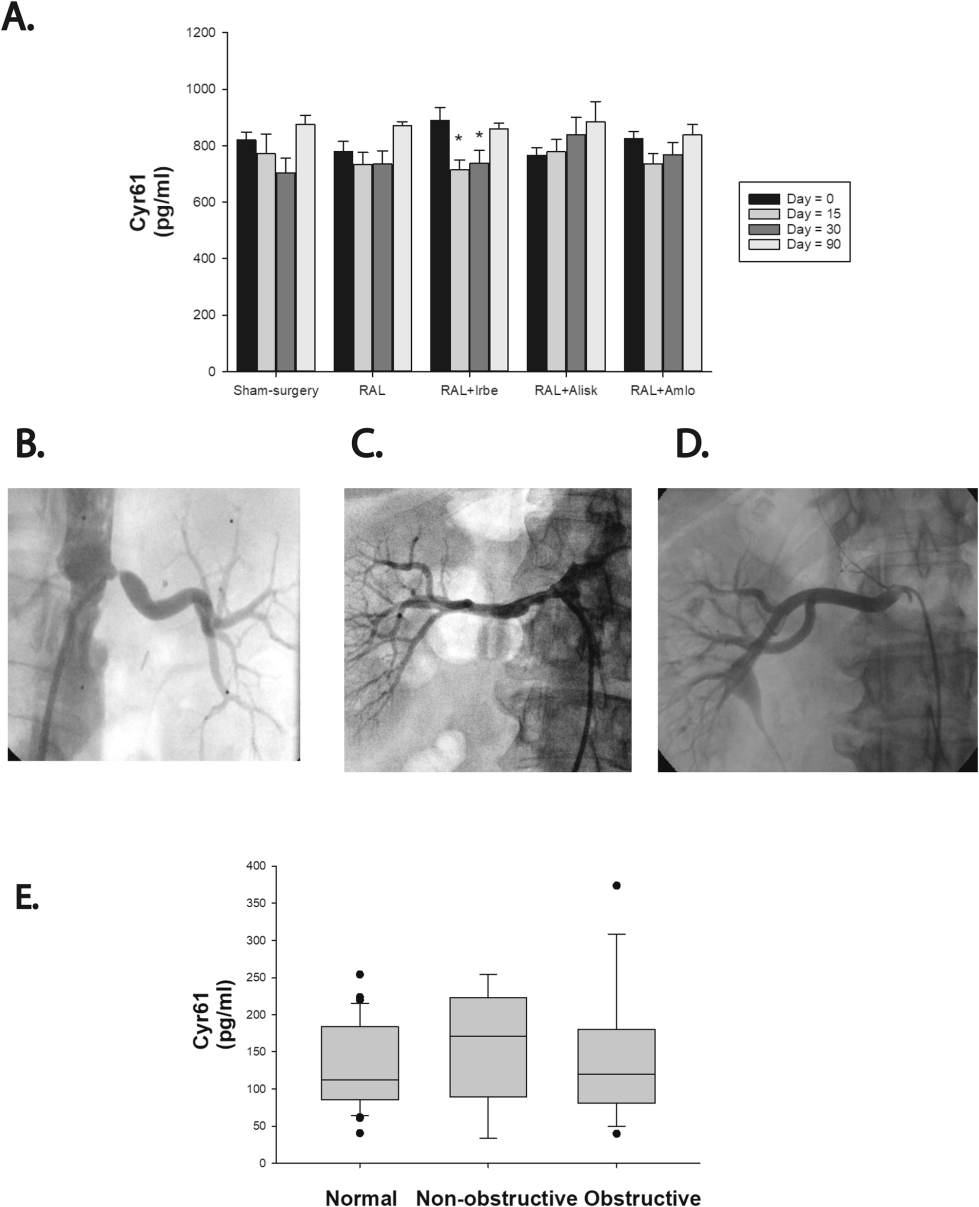
Serum levels of Cyr61 in mice and humans. Serum levels of Cyr61 in mice with unilateral renal artery ligation were determined from tail vein blood immediately prior to surgery and then at 15, 30 and 90 days after surgery (**A**). Representative images of a renal angiogram from a patient with obstructive RAS (**B**), non-obstructive RAS (**C**) or normal renal arteries (**D**). Serum levels of Cyr61 in each group as determined by a commercially available ELISA assay (**E**).

While previous studies reported that unilateral RAL in mice resulted in a rapid increase in renal production of Cyr61 with a peak at 6–9 h^23,24^, it is unclear whether there is a sustained increase in serum levels. In the current model, serum levels of Cyr61 were not elevated when measured 15, 30 and 90 days after RAL. Specifically, there was no increase in Cyr61 in mice with RAL compared to mice who underwent sham-surgery at any time point or in the groups of mice with RAL treated with amlodipine or aliskiren (Fig. 4A). Irbesartan treatment had minimal effects at days 15 and 30 that were statistically significant.

Clinical studies have shown that serum-levels of Cyr61 are elevated in patients with coronary artery disease^25–27^, pulmonary hypertension^28^, acute heart failure^29^, and systemic lupus erythematosus^30^. To determine whether serum levels of Cyr61 were increased in patients with RAS, 55 patients who had renal artery assessment were divided into obstructive RAS (≥ 60% stenosis; n = 14; Fig. 4B), non-obstructive RAS (10–60% stenosis; n = 9; Fig. 4C) and normal renal arteries (n = 32; Fig. 4D). The mean age was 69 ± 12 years, 64% were males, 20% were African Americans and major comorbidities included diabetes mellitus (44%), hypertension (89%) and obstructive coronary artery disease (58%; Table 1). Results showed that there was no difference in serum-CYR61 levels between the 3 groups (Fig. 4E).

**Table 1:** Demographics of patients.

	No RAS	RAS < 60%	RAS ≥ 60%	P value
n	32	9	14	
Age (years)	66.9 ± 12.2	71.1 ± 14.3	71.5 ± 10.6	0.42
Male (%)	22 (69%)	5 (56%)	8 (57%)	0.65
African American	7 (22%)	2 (22%)	2 (14%)	0.83
BMI	29.6 ± 6.2	37.9 ± 4.5	29.6 ± 4.3	0.95
DM	15 (47%)	3 (33%)	6 (43%)	0.77
HTN	27 (84%)	9 (100%)	13 (93%)	0.72
Creatinine	1.04 ± 0.38	1.14 ± 0.36	1.32 ± 0.55	0.17
HCT	39.8 ± 5.2	37.9 ± 4.5	36.8 ± 3.8	0.14
SBP	142.0 ± 23.9	138.2 ± 15.1	152.6 ± 31.2	0.59
DBP	74.9 ± 15.3	78.0 ± 22.1	70.1 ± 18.3	0.55
Obstructive CAD	16 (50%)	6 (67%)	10 (71%)	0.34
CYR61	129.4 ± 56.1	154.2 ± 76.2	141.9 ± 87.6	0.70

There are no data on the relationship between hypertension and Cyr61 expression in humans and thus we examined the correlation between systolic blood pressure and serum-levels of Cyr61 in our patient population. We found no correlation between Cyr61 serum levels and systolic blood pressure (*p* = 0.41) nor we did find any difference in Cyr61 in patients with systolic blood pressure ≤ 140 mm Hg (n = 31) vs systolic blood pressure > 140 mm Hg (n = 24) (122.6 [81.3, 186.1] pg/ml vs 134.2 [89.5, 182.2] pg/ml; *p* = 0.79). These results must be interpreted with caution as serum Cyr61 levels and systolic BP were measured at a single time point and, given the limited sample size, the analysis could not control for potential confounders such as age, gender, co-morbidities or the effect of anti-hypertensive medications.

## Discussion

This study provides evidence that Cyr61 is expressed in atherosclerotic plaques from 18-week old male ApoE^−/−^ mice with RAL and that expression is reduced by medications that lower blood pressure. Treatment with irbesartan, an AT1 receptor blocker, or aliskiren, a renin inhibitor, had effects similar to those observed with amlodipine, a calcium receptor blocker, on lipid deposition in the aortic arch, lipid deposition in the suprarenal descending aorta and Cyr61 expression in atherosclerotic plaques in the infrarenal aorta. Results showing a reduction in Cyr61 expression within atheroma by medications which reduce blood pressure via three separate mechanisms strongly suggest that blood pressure, rather than activation of the renin-angiotensin system, is a more powerful regulator of aortic expression of Cyr61 in ApoE^−/−^ mice with partial unilateral RAL.

The current study adds to accumulating evidence that Cyr61 expression is upregulated at sites of vascular injury and atherosclerosis. Numerous studies have shown minimal expression of Cyr61 in normal arteries but markedly increased expression at sites of atherosclerosis or vascular injury including carotid endarterectomy specimens^6,7^, aorta in older ApoE^−/−^ mice^6,9^, and at sites of mechanical or drug-induced vascular injury^8,10–13,31^. There is also data that Cyr61 is functionally important in vascular injury responses as inhibition of Cyr61 using siRNA significantly suppressed neointimal hyperplasia at days 14 and 28 after injury^10^ and constitutively active FOXO3a gene transduction suppressed CYR61 expression and neointimal formation after balloon injury with the reduction in neointimal hyperplasia being reversed by Cyr61 replenishment^11^.

Serum levels of Cyr61 in mice were not increased 15, 30 or 90 days after RAL suggesting that chronic renal ischemia is not a stimulus for Cyr61 release. This is in contrast to the acute phase of renal ischemia where animal experiments have shown that the proximal straight tubules produce Cyr61 within a few hours after onset of renal ischemia with peak effect at 6–9 h^23,32^. Factors that regulate serum levels of Cyr61 have not been identified but, in addition to being produced in the kidney^33^, Cyr61 is produced by endothelial and epithelial cells, vascular smooth muscle cells, fibroblasts, monocytes, activated platelets and cardiomyocytes. Numerous stimuli which increase production of Cyr61 in cell culture have been identified including growth factors, thrombin, hypoxia, disturbed shear stress and mechanical stretch^34^. In a murine model, myocardial Cyr61 expression was markedly increased by ischemia and reperfusion^35^.

The current study is the first to examine serum Cyr61 levels in patients with RAS. Dividing patients into three groups, obstructive RAS (≥ 60% stenosis), non-obstructive RAS (10–60% stenosis) and normal renal arteries, we found no difference in Cyr61 levels in a population with high rates of diabetes mellitus, hypertension and obstructive coronary artery disease. Previous studies have shown that Cyr61 is elevated in patients with ST elevated myocardial infarction (STEMI) compared to patients with non-STEMI^35^, patients with acute coronary syndromes compared to patients with stable angina or patients without coronary artery disease^27^, patients hospitalized with acute heart failure compared to age and gender matched controls^36^ and patients with idiopathic or connective tissue associated pulmonary arterial hypertension compared to age, gender and disease state matched controls^28^. A role for Cyr61 as a prognostic biomarker has been suggested by some investigators based on data showing that Cyr61 improved risk stratification for all-cause mortality in patients with acute coronary syndromes when added to the Global Registry of Acute Coronary Events (GRACE) risk score at 30 days and 1 year^26^ and that Cyr61 levels were predictive of 6-month mortality in patients with acute heart failure^36^. Several studies have shown that serum levels of Cyr61 are not affected by chronic renal disease^29,36^.

Results of the current study showed that treatment with irbesartan, aliskiren or amlodipine reduced Cyr61 expression in aortic atheroma at doses that decreased blood pressure and reduced the accelerated lipid deposition in the aortic arch and descending aorta associated with chronic RAL in ApoE^−/−^ mice. While Ang-II levels were transiently increased in this model, it is unlikely that Ang-II is the sole stimulus for Cyr61 production in atheroma as amlodipine reduced Cyr61 levels similarly to irbesartan and aliskiren. Nussberger et al. previously showed differential effects of amlodipine, aliskiren and irbesartan on aortic plaque stabilization in the 2 K,1C model in ApoE^−/−^ mice but at aliskiren and irbesartan doses that were fivefold and tenfold, respectively higher than used in the current study^20^. Additional studies are necessary to further characterize mechanisms stimulating Cyr61 production in the aorta in ApoE^−/−^ mice.

There are several limitations of our study. One is that we only looked at a single time point (90 days after surgery) and thus have not established the kinetics of Cyr61 expression in ApoE^−/−^ mice with RAL. Second, these studies were not designed to examine the effects of Cyr61 on the vascular wall and have not shown that Cyr61 contributes to the progression of atherosclerosis. Lastly, only 42% of patients had blood samples obtained at the time of renal angiography. It is unlikely that there was significant progression of renal artery disease in the 58% of patients who had blood samples obtained within 6 months of renal artery assessment as Crowley et al. found that only 11.1% of patients had progression of renal artery disease during an average followup of 2.6 ± 1.6 years^37^.

In summary, these studies show that there is significant expression of Cyr61 within the descending aorta of ApoE^−/−^ mice with surgically-induced RAL compared to minimal expression in mice that underwent sham surgery. Treatment of mice with RAL with irbesartan, aliskiren or amlodipine resulted in significant decreases in blood pressure, lipid deposition in the aorta and Cyr61 expression in aortic atheroma. Serum levels of Cyr61 in mice were not elevated when measured 15, 30 and 90 days after RAL. There were no differences in serum levels of Cyr61 between groups of patients with angiographically normal renal arteries, those with RAS < 60% and those with RAS ≥ 60%. Taken together these data demonstrate that Cyr61 is increased in atheroma in ApoE^−/−^ mice with RAL and is reduced by anti-hypertensive medications although a mechanistic role has not been established. Serum levels are unlikely to provide any useful prognostic information in patients with unilateral renal artery stenosis.

### Human subjects/informed consent statement

All patients enrolled in this study gave informed consent.

### Ethical approval

All procedures followed were in accordance with the ethical standards of the responsible committee on human experimentation (institutional and national) and with the Helsinki Declaration of 1975, as revised in 2000 (5). Informed consent was obtained from all patients for being included in the study. All institutional and national guidelines for the care and use of laboratory animals were followed and approved by the appropriate institutional committees..
